# Construction of PPSU-MoS_2_/PA-MIL-101(Cr) Membrane with Highly Enhanced Permeance and Stability for Organic Solvent Nanofiltration

**DOI:** 10.3390/membranes12070639

**Published:** 2022-06-21

**Authors:** Qin Liu, Xing Wu, Zongli Xie, Kaisong Zhang

**Affiliations:** 1 College of Harbour and Coastal Engineering, Jimei University, Xiamen 361021, China; liuhy519@163.com; 2 Key Laboratory of Urban Pollutant Conversion, Institute of Urban Environment, Chinese Academy of Sciences, Xiamen 361021, China; 3 CSIRO Manufacturing, Private Bag 10, Clayton South, VIC 3169, Australia; xing.wu@csiro.au (X.W.); zongli.xie@csiro.au (Z.X.)

**Keywords:** organic solvent nanofiltration, MIL-101(Cr), polyamide, composite membrane

## Abstract

Membranes with excellent separation performance and stability are needed for organic solvent nanofiltration in industrial separation and purification processes. Here we reported a newly PPSU-MoS_2_/PA-MIL-101(Cr) composite membrane with high permeance, good selectivity and stability. The MIL-101(Cr) was introduced in the polyamide (PA) layer via the PIP/TMC interfacial polymerization process on a microporous PPSU-MoS_2_ substrate. At a small doping amount of 0.005 wt% MIL-101(Cr), the PPSU-MoS_2_/PA-MIL-101(Cr) composite membrane exhibited a high methanol permeance of 12.03 L m^−2^ h^−1^ bar^−1^, twice higher than that of the pristine membrane without sacrificing selectivity. Furthermore, embedding MIL-101(Cr) notably enhanced the stability of the composite membrane, with permeance only decreasing by 8% after a long time operation of 80 h (pristine membrane decreased by 25%). This work demonstrated a composite membrane modified by MIL-101(Cr) with superior separation performance, which provides potential application of MOF materials for high-performance membranes in organic solvent nanofiltration and a theoretical foundation for future research in studying MOF’s influence on membrane properties.

## 1. Introduction

Organic solvents are broadly applied in varied industries associated with pharmaceutical, petrochemical, food and textiles. Commonly, most organic solvents including alcohols, ketones, esters and hydrocarbons are toxic or carcinogenic. Therefore, the harmful and costly organic solvents must be recovered by energy-efficient and cost-effective techniques. Organic solvent nanofiltration (OSN) with membranes has ignited remarkable interest in this field for its superior separation efficiency and low energy consumption [1].

The thin-film composite (TFC) membranes comprised of an ultrathin selective layer and a porous substrate have been extensively studied in solvent treatment due to their convenient fabrication approaches and easy tuning feature for the target application. Various polymers such as Polyacrylonitrile (PAN) [2,3,4,5], Polyimide (PI) [6,7], Polysulfone (PSf) [8,9] and Polyphenylsulphone (PPSU) [10,11] are employed as substrates due to their low permeate resistance. The selective layer is generally formed by the interfacial polymerization method, which is carried out by two monomers dissolved in two immiscible solutions: diamine aqueous and acyl chloride organic phases. The cross-linked polyamide (PA) film formed at the interface of these two immiscible solutions generates excellent selectivity and high solvent permeance. With the aim of increasing membrane permeability and stability toward the target industry application, hybridizing membranes by including nanoparticles to construct thin-film nanocomposite (TFN) membranes has been experimentally and theoretically demonstrated. Various nanoparticles have been employed as additives in TFN membranes, such as silver [12], carbides [13,14], molybdenum disulfide [15] and titanium dioxide [16]. Recently, the metal-organic frameworks (MOFs) [17,18] have triggered extensive efforts to modify separating membranes by virtue of specific favors. For example, MOFs have extremely high specific surface area related to the internal porous structure, which provides a huge number of transport pathways for solvent molecules, resulting in the great improvement of membrane permeability. Moreover, the presence of organic ligands part within MOFs can enhance their compatibility with the polymer matrix [19]. 

Amongst MOFs, MIL-101(Cr) has been extensively studied in the fields of carbon dioxide capture, treatment of harmful components, and catalytic reactions [20,21,22,23] due to its high surface area, large pore volume, good hydrophilicity and chemical stability. In addition, the specific pore size (1.2 and 1.6 nm) and high porosity (0.83) enable MIL-101(Cr) an ideal candidate for membrane separation at the molecular level [24,25]. Song and co-authors [26] blended polyamide with MIL-101(Cr)@GO to fabricated an RO membrane based on the PES substrate. With a doping amount of 0.01 wt%, water flux of the membranes can be increased by 85.21%, along with slightly improved NaCl rejection. Later, they continued to employ MIL-101(Cr) to prepare PA composite nanofiltration membranes using the PSf-based substrate, in which the 0.3 wt% loading of MIL-101(Cr) endows the composite membrane with a significant increase in water flux, improving from 43.66 to 63.36 L m^−2^ h^−1^ under a pressure of 1 Mpa [27]. Xu and co-authors [28] used MIL-101(Cr) as the additive to prepare a TFN membrane for water desalination, MIL-101(Cr) nanoparticles increased water permeance by 44% compared to undoped PA membranes. However, the research on blending PA with MIL-101(Cr) for high-performance membranes towards OSN applications is rarely reported, and there is a lack of study on the mechanism exploration of MIL-101(Cr) for the improvement of membrane separation performance and the influence of MIL-101(Cr) on physicochemical properties of membranes. Thereby, the development of TFN membrane incorporated with MIL-101(Cr) and the systematic investigation of the interaction between polymer matrix and the additives are needed. 

**Figure 1:**
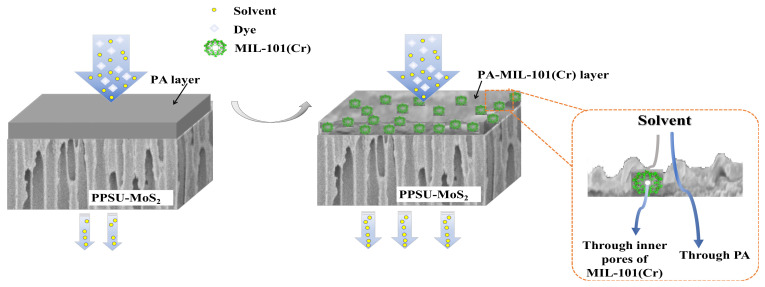
Mechanism of blending PA with MIL-101(Cr) for superior OSN membrane.

In this study, MIL-101(Cr) nanoparticles were fabricated and employed as nano-additives to develop TFN membranes for OSN application. The PA film was fabricated via the interfacial polymerization method using PPSU-MoS_2_ as a support layer. Here the employed substrate could provide superior solvent permeance with high porosity. MIL-101(Cr) nanoparticles were determined by FTIR, EDX, XRD, TEM, TGA and BET analysis, and the physicochemical properties and OSN performance of fabricated membranes were systematically studied to understand the effect of MIL-101(Cr) on the separation efficiency, dye retention, stability, solvent permeance and transmission mechanism of the TFN membranes. Figure 1 illustrates the mechanism of MIL-101(Cr) incorporated PA for a superior OSN membrane.

## 2. Experimental Section 

### 2.1. Materials and Reactants

PPSU (P3010) was purchased from BASF Co., Ltd., MoS_2_, terephthalic acid, chromium nitrate-9-water (Cr(NO_3_)_3_·9H_2_O), 1-methyl-2-pyrrolidinone (NMP), hydrofluoric acid (HF), piperazine (PIP), trimesoyl chloride (TMC), sodium lauryl sulfate (SLS), (±)-Camphor-10-sulfonic acid (CSA), triethylamine (TEA), hexane, methanol, ethanol, isopropanol (IPA), acetonitrile, ethyl acetate, methyl orange (MO), crystal violet (CV), bromothymol blue (BTB) and rose bengal (RB) were purchased from Sigma-Aldrich. All chemicals mentioned in this work were analytical reagents and utilized without further purification.

### 2.2. Synthesis of MIL-101(Cr)

The synthesis of MIL-101(Cr) was followed by the method previously reported by Férey and co-authors [29]. Briefly, 0.6 mL of HF, 500 mg of terephthalic acid and 1.2 g of Cr(NO_3_)_3_·9H_2_O were mixed with 15 mL of distilled water to form a homogeneous reaction solution and heated at 210 °C for 8 h. After naturally cooling down, the precipitated MIL-101(Cr) can be obtained following the filtration. The solid product with green colour was successively neutralized with hot dimethylformamide, washed with ethanol and dried overnight in an oven at 75 °C.

### 2.3. Preparation of PPSU-MoS_2_ Substrate

The PPSU-MoS_2_ substrate membranes were prepared by the phase conversion method as follows.A total of 0.25 wt% of MoS_2_ and 16 wt% of PPSU was mixed in NMP, and stirring was maintained until an even casting solution was formed. The uniform casting solution was coated onto a polyester non-woven fabric via a knife blade with a casting gap of 200 µm. Afterward, the PPSU-MoS_2_ substrate can be formed after being immersed in the DI water. The porosity of fabricated PPSU-MoS_2_ substrate is 0.824 with methanol permeance above 3000 L m^−2^ h^−1^ bar^−1^.

### 2.4. Preparation of PPSU-MoS_2_/PA-MIL-101(Cr) Membrane

The PPSU-MoS_2_/PA-MIL-101(Cr) membranes were prepared by the interfacial polymerization method according to the previous report [9]. Typically, the organic phase was prepared by dissolving 0.35 g of TMC in 100 ml of hexane, in which the nanoparticle MIL-101(Cr) ranging from 0.00 w/v% to 0.02 w/v% was uniformly dispersed by ultrasonic, and the specific doping amount of MIL-101(Cr) was listed in Table 1. The aqueous phase containing 1.6 wt% of PIP, 0.1 wt% of SDS, 1.5 wt% of TEA and 1.5 wt% of CAS was prepared and poured on the PPSU-MoS_2_ substrate. Then, the above solution was poured out from the substrate after 45 s, and any residual solution droplets were removed through tissue papers. The organic phase was poured on the substrate subsequently to allow PIP to react with TMC, and then the membrane was moved to the oven at 60 °C after 20 s of the IP process for further cross-linking reaction. The fabricated composite membranes were named M0, M1, M2, M3, M4, M5 and M6, corresponding with various doping amounts of MIL-101(Cr) (Table 1). 

### 2.5. Characterization of MIL-101(Cr)

The chemical structure and elemental composition of MIL-101(Cr) were analyzed by Fourier infrared spectroscopy (FTIR) using a NicoletiS10 spectrometer (Thermo, iS10, Thermo, Waltham, MA, USA) and X-ray scanning energy spectroscopy (EDX-PV77-47230), respectively. Pore size distribution of MIL-101(Cr) was evaluated by N_2_ isotherms at 77k using a multiple point Brunauer Emmet Teller (BET) (ASAP 2420M+C, Micromeritics, Duluth, MN, USA). The XRD spectrum of the MIL-101(Cr) was characterized using an X-ray diffraction facility (X’Pert PRO, PANalytical B.V. Holland) with the range of 2θ = 2 to 80°. The morphology and thermal stability of MIL-101(Cr) were measured by the transmission electron microscopy (TEM) via an H-7650 (HITACH, Tokyo, Japan) instrument and thermogravimetric analysis (TGA) facility (Perkin Elmer, Waltham, MA, USA), respectively. 

### 2.6. Characterization of Membranes

Field emission scanning electron microscopy (FESEM, Hitachi S-4800, Tokyo, Japan) and transmission electron microscopy (TEM, H-7650, HITACH, Tokyo, Japan) were utilized to observe the surface and cross-sectional morphology of membranes. Atomic force microscopy (AFM, Dimension 3100, Bruker, WI, USA) was employed to determine the surface roughness of membranes. X-ray photoelectron spectroscopy (XPS, Axis Supra, Kratos, Manchester, UK) was applied to investigate the atomic composition of membranes. 

### 2.7. Density and Cross-linking Degree of Membranes 

**Figure 2:**
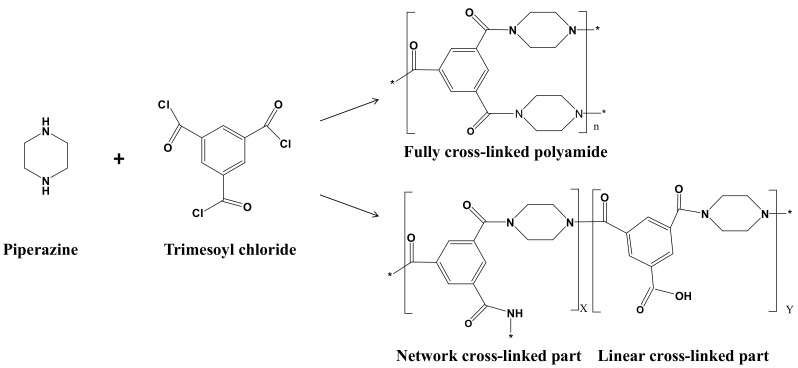
The IP process between PIP and TMC.

The density of membranes depends on the cross-linking degree of the PA, which can be calculated based on the element ratio of *O* and *N* from XPS measurement [30]. Figure 2 presents the cross-linked network formed by the IP process between PIP and TMC. The degree of network cross-linking (*DNC*) was calculated according to the following Equations (1) and (2).

(1) $$\frac{O}{N}=\frac{3X+4Y}{3X+2Y}$$

(2) $$DNC(\%)=\frac{X}{X+Y}\times 100\%$$

where *X* represents the network cross-linking structure in the polyamide, and *Y* refers to the linear structure in the polyamide.

### 2.8. Characterization of Membrane Performance

The OSN performance of membranes was performed using a dead-end filtration facility (HP4750, Sterlitech, America) with an effective membrane area of 14.6 cm^2^. The dye/alcohol solution with a dye concentration of 20 ppm was prepared as the feed solution, and the filtration measurement was conducted under pressure of 4 bar at room temperature. During filtration, the feed solution was continuously stirred at a speed of 500 rpm to minimize the concentration polarization. Permeance and dye rejection of the membrane were calculated according to Formulas (3) and (4), respectively.

(3) $$P=\frac{\Delta m}{\rho_{s}\times A\times \Delta p\times \Delta t}\;$$

where P is the permeance of organic solvent, ∆m refers to the weight of the permeating solvent collected during a certain time (∆t), ρ_s_ refers to the density of organic solvent, A represents the effective membrane area and ∆p represents the trans-membrane pressure.

(4) $$R\left(\%\right)=\left(1-\frac{C_{p}}{C_{f}}\;\right)$$

where R is the solute rejection of membrane, C_p_ and C_f_ refer to the solute concentration in the permeate and feed solution, respectively. The concentration of solute in organic solvent was analyzed by a UV-vis spectrophotometer (UV-2450, Shimadzu, Kyoto, Japan).

## 3. Results and Discussion

### 3.1. MIL-101(Cr) Characterization

**Figure 3:**
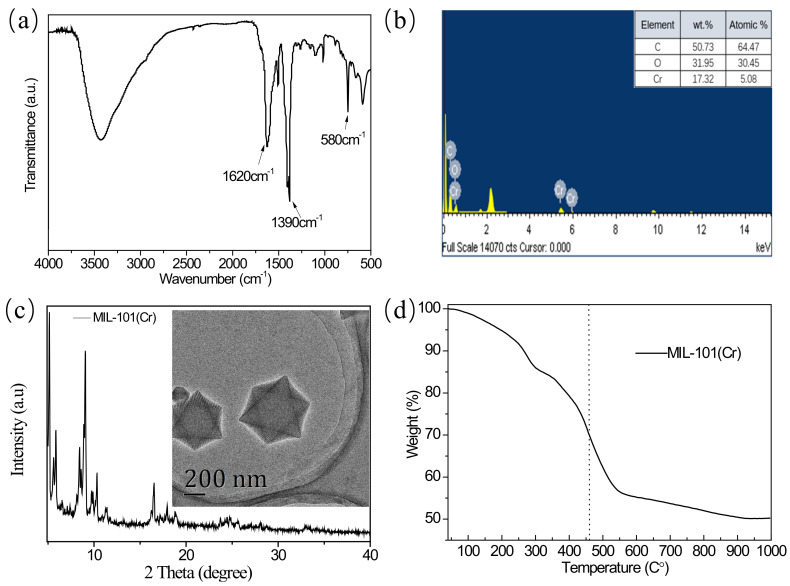
(**a**)The FTIR spectra of MIL-101(Cr); (**b**)the EDX analysis of MIL-101(Cr); (**c**)the XRD spectra and TEM image of MIL-101(Cr); (**d**)the TGA curve of MIL-101(Cr).

Figure 3a presents the FTIR result of MIL-101(Cr). The absorption peak at 1620 cm^−1^ corresponds to the asymmetric stretching vibration of the carboxyl group, and the peak at 1390 cm^−1^ belongs to the symmetric stretching vibration of the carboxyl group [31], indicating the presence of dicarboxylate in the MOF framework. The peak at 580 cm^−1^ refers to the vibration absorption of Cr-O in MIL-101 (Cr) [32]. The presence of Cr was confirmed by EDX measurement (Figure 3b). The diffraction peaks at 5° and 9° in XRD results (Figure 3c) are consistent with previous reports on confirming the crystal structure of the MIL-101(Cr) [32]. TEM morphology (inset picture in Figure 3c) unveiled approximate octahedral crystals of porous MIL-101(Cr) with uniform size, which proved the successful synthesis of MIL-101 (Cr). Figure 3d shows the TGA curve of MIL-101(Cr). There are two distinct weight loss stages: weight loss before 300 °C was caused by the evaporation of water adsorbed on the surface of MIL-101(Cr); the mass loss around 460 °C can be ascribed to the decomposition of the organic linkage fraction within MIL-101(Cr). 

**Figure 4:**
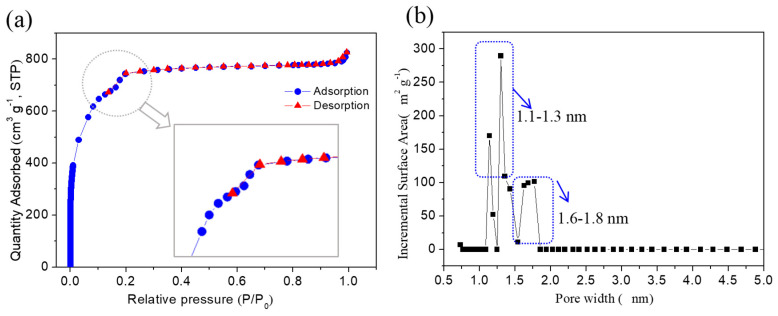
(**a**) The N_2_ adsorption and desorption curves of MIL-101(Cr) and (**b**) pore size distribution.

The N_2_ adsorption and desorption curves of MIL-101(Cr) in Figure 4a demonstrate an obvious type-I N_2_ isotherm. The adsorption amount of N_2_ increases rapidly under low pressure of P/P_0_ = 0~0.1, indicating the nanoporous structure of MIL-101(Cr) [33]. Curve variation at P/P_0_ = 0.1 and P/P_0_ = 0.2 corresponds to the bigger and smaller porous cage structures in MIL-101(Cr) [34], respectively; the pore size distribution of MIL-101(Cr) in Figure 4b confirms these two windows’ sizes of 1.1~1.3 and 1.6~1.8 nm, respectively.

### 3.2. Characterization of Membranes

**Figure 5:**
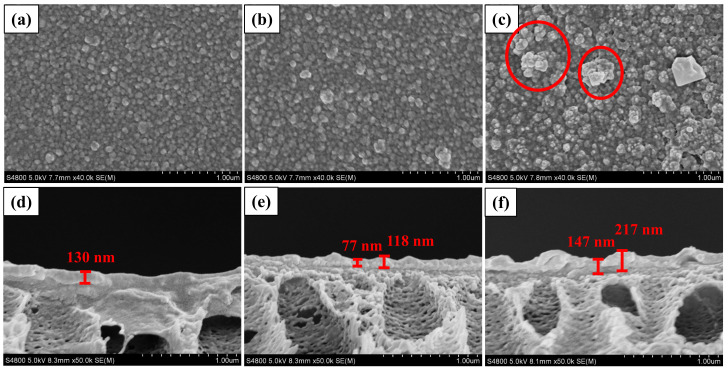
SEM pictures of the surface and cross-section: (**a**,**d**) M0, (**b**,**e**) M3, (**c**,**f**) M6.

**Figure 6:**
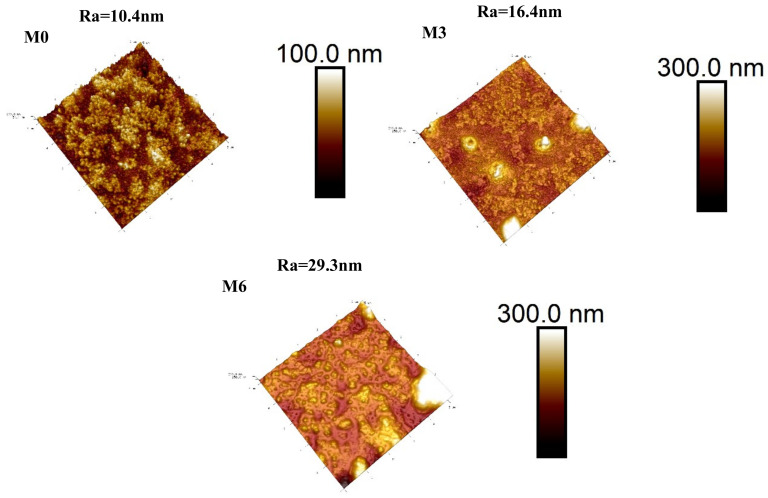
AFM pictures of the membrane surfaces of M0, M3, M6.

SEM analysis was performed to study the influence of MIL-101(Cr) on membrane morphology. Figure 5 shows the surface and cross-sectional morphology for membranes M0, M3 and M6. All membranes exhibited typical convex structures that result from the fast interfacial polymerization between PIP and TMC [35,36,37]. M0 gives a relatively smooth polyamide layer, while M3 and M6 exhibit rougher top layers with many protrusions arising from the MIL-101(Cr) wrapped by polyamide, as shown in the red circled area in Figure 5c. Further comparing Figure 5b, c and 5e, f, membrane M3 with lower MIL-101(Cr) embedding (0.005 w/v%) was flatter and had smaller protrusions than membrane M6 with higher addition of MIL-101(Cr) (0.01 w/v%). This was because MIL-101(Cr) is prone to agglomeration at a higher concentration, which consequently caused a large convex structure on the surface of the PA layer, as well as a rougher surface for the membrane M6. AFM analysis confirmed the increase in surface roughness after adding MIL-101(Cr), which increased from Ra = 10.4 to Ra = 29.3 nm (Figure 6). The reason for the above difference between TFC and TFN membranes can be described as the impact of MIL-101(Cr) on the IP process. During the interfacial polymerization, PIP diffuses into the oil phase and reacts with TMC to form the PA with cross-linked network structures. It has been reported that the diffusion rate of the diamine monomer can directly affect the cross-linking degree of the reaction and the thickness of the polyamide layer [38]. The introduction of MIL-101(Cr) nanoparticles can hinder the diffusion of PIP into the oil phase to react with TMC and interfere with the formation process of the PA chain at the interface between PIP and TMC. Moreover, the thickness of the pristine membrane was around 130 nm, and the M3 with the low addition of MIl-101(Cr) resulted in a thinner top layer (from 77 to 118 nm), which will contribute to the fast transmission of solvents through M3. 

**Figure 7:**
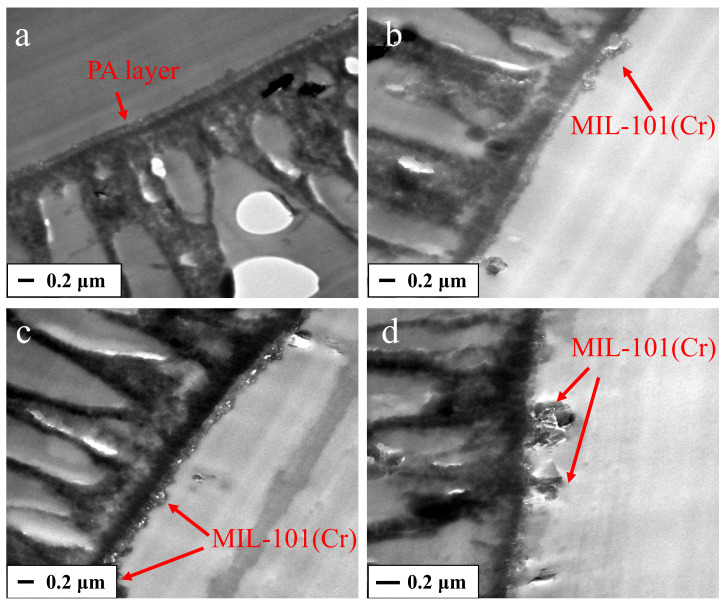
TEM images for the cross-section of pristine TFC membrane (**a**) and 0.005 wt% MIL-101(Cr)-TFN membrane (**b**–**d**).

The dispersion of inorganic nanoparticles in the PA films was observed by TEM measurement. Because the incorporated inorganic nanoparticles and polymer matrix could exhibit obvious morphology differences under the electron beam, inorganic fillers generally presented easily discernable dark and specific shapes. Figure 7 displays the cross-sectional images of the membranes M0 and M3. Obviously, the pristine membrane M0 (Figure 7a) presents a relatively flat and smooth top layer, which is the typical structure formed by PIP and TMC interface polymerization. In Figure 7b–d, the MIL-101(Cr) can be clearly observed with its discerned dark and specific shape, demonstrated the successful incorporation of MIL-101(Cr) into the PA layer. Combined with the SEM results, it can be concluded that MIL-101 (Cr) can be incorporated into the polyamide layer via the IP process. 

The atom ratio of O/N represents the cross-linking degree of the polyamide layer, which ranges from 1.0 to 2.0. When the value of O/N ratio is 1.0, it indicates the polyamide layer is theoretically completely cross-linked; that is, all of the O and N atoms are combined by the amide groups. On the other hand, the 2.0 O/N ratio suggests a polyamide layer with a completely linear structure [39,40]. The O/N ratios of the polyamide layer of prepared NF membranes were determined by XPS, and the results are shown in Table 2. It should be pointed out that MIL-101 (Cr) also contains oxygen atoms; hence, the O/N ratio in polyamide must exclude the oxygen from MIL-101 (Cr), which had been detected via the EDX analysis. Thus, the cross-linking degree of PA was calculated according to the O/N ratio in PA, which had been referred as O/N^b^. According to the results presented in Table 2, the O/N^b^ ratio of the PA layer increased from 1.223 to 1.335 after the incorporation of MIL-101 (Cr), and the cross-linking degree of the PA layer correspondingly decreased from 69.9% to 56.92%. This hinted that MIL-101 (Cr) hinders the interfacial polymerization and reduces the formation of the cross-linking network in PA.

### 3.3. Organic Solvent Nanofiltration Performance of Membranes

**Figure 8:**
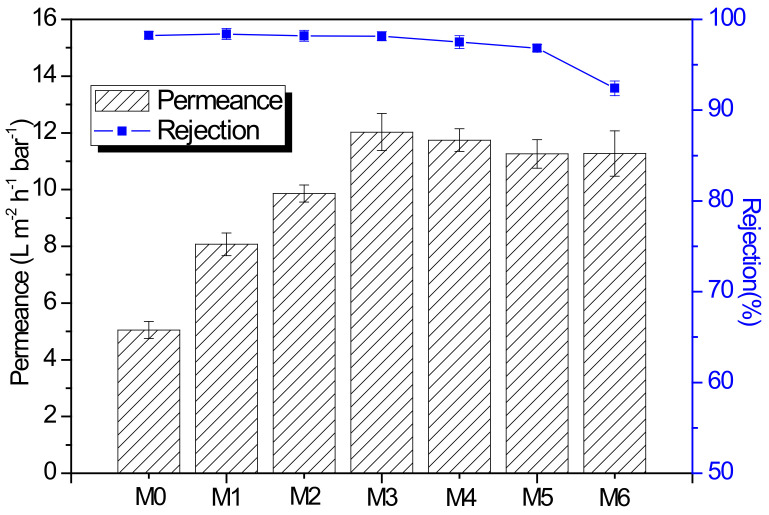
Methanol permeance and RB rejection of PA and PA-MIL-101(Cr) membranes.

The organic solvent nanofiltration test was carried out under the pressure of 4 bar, and the 20 ppm RB/methanol solution was prepared as the feed. As illustrated in Figure 8, the permeance of the composite membrane with MIL-101(Cr) was significantly increased compared with the pristine membrane. When the concentration of MIL-101(Cr) is 0.005 wt%, membrane M3 achieved the highest permeance of 12.03 L m^−2^ h^−1^ bar^−1^ without sacrifice selectivity (RB rejection is 98.15%), which improved more than two times compared with the unmodified membrane M0 (5.05 L m^−2^ h^−1^ bar^−1^). This high enhancement of solvent permeance for membrane M3 can be ascribed to the following reasons. First, MIL-101(Cr) has a large number of hydrophilic carboxyl and amide groups, which can promote the adsorption of alcohol molecules on the surface of MIL-101(Cr), so that alcohol molecules can quickly pass through the additional transmission channels provided by porous MIL-101(Cr). In addition, the addition of MIL-101(Cr) can promote the penetration of alcohol molecules by reducing the solvent transmission resistance in the void area between nanoparticles and the polyamide matrix. Moreover, the increased roughness of the membrane surface can provide a larger area for the transportation of alcohol molecules, resulting in an increase in solvent permeance. However, when the concentration of MIL-101(Cr) is above 0.005 wt%, membrane selective performance decreases quickly with the increase of MIL-101(Cr) concentration. This is because particle agglomeration is prone to be formed with a high concentration, which will cause defects in the polyamide layer. Furthermore, the addition of MIL-101(Cr) could hinder the interfacial polymerization process and reduce the cross-linking density of the polyamide layer (proved by the results in Table 2). 

**Figure 9:**
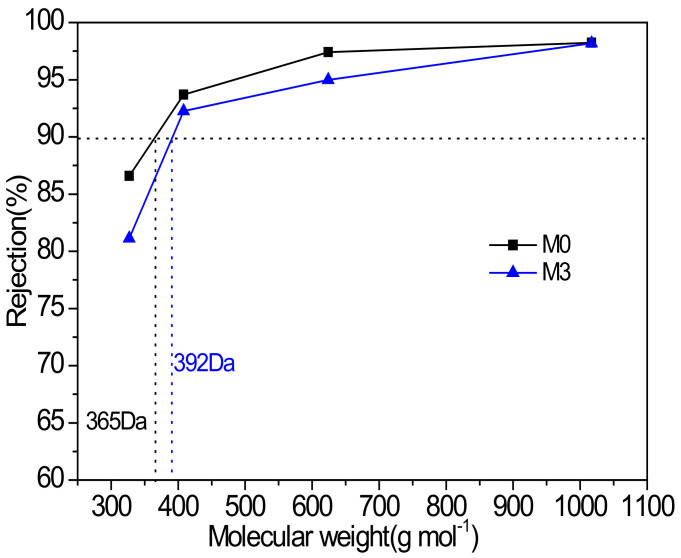
Rejection performance of the PA and PA-MIL-101(Cr) membranes against various dyes (dye concentration: 20 ppm in methanol).

The molecular weight cut-off (MWCO) of membranes was evaluated using various dyes with different molecular weights ranging from 300 to 1000 g mol^−1^ as solutes. The characteristics of different dyes are listed in Table 3. Figure 9 gives the results examined by RB, BTB, CV and MO in methanol. The MWCO of membrane M3 (392 Da) was appreciably higher than that of the M0 membrane (365 Da), which was related to the influence of MIL-101(Cr) on the polyamide depicted above. 

**Figure 10:**
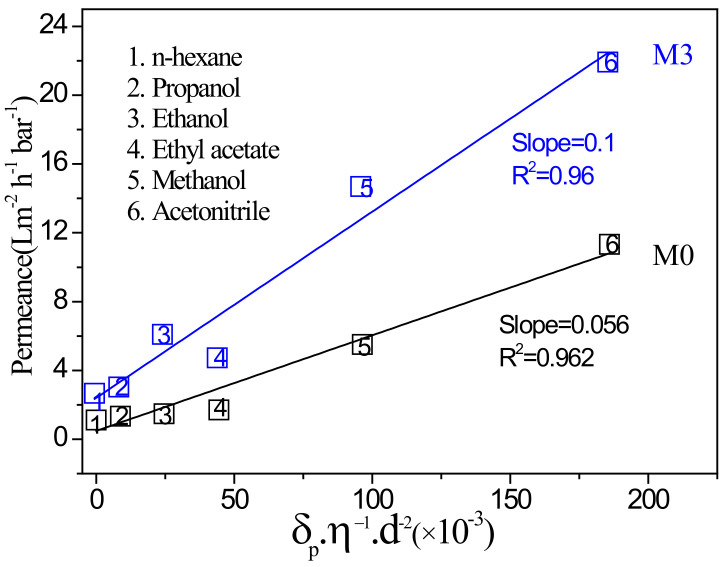
Plot of solvent permeances against the combined solvent property (viscosity, molar diameter, and solubility parameter) for M0 and M3 membranes.

The relationship between solvent permeance of the membrane and the physical parameters of solvent molecules is crucial for studying the solvent molecule transfer mechanism of modified polyamide membranes. The physical parameters of solvent molecules, including solubility parameters (δ, MPa^1/2^), viscosity (η, cP) and molar diameter (d, nm), are decisive factors that may affect solvent permeance. At present, there are two main models for describing the solvent transport mechanism in the membrane, which are the dissolution-diffusion model and the pore model. Most dense polymers conform to the dissolution-diffusion model, and most inorganic membranes are consistent with the pore model. The dissolution-diffusion model is briefly described as the solvent must dissolve on the surface of the membrane and pass through the membrane by diffusion. The model can be described by P = K_1_δ/ηd, in which K_1_ is the proportional constant (m^3^ Pa^−0.5^), the Hansen solubility parameter *δ* of the organic solvent reflects the intermolecular interaction, *d* is the molecular size of the solvent, *η* is the viscosity of the solvent, and *d* and *η* affect the diffusion rate of the solvent. Herein, several varied organic solvents were employed to examine the permeance of membranes M0 and M3. These organic solvents and related physical parameters are listed in Table 4. In Figure 10, a positive correlation between solvent permeance and the δ/ηd parameter of the solvent could be observed for both membrane M0 and M3, which is completely in agreement with the previously report for polyamide membrane fabricated by m-Phenylenediamine (MPD) and TMC [30]. This indicates that the addition of MIL-101 (Cr) does not change the solvent transport model of the polyamide membrane. Furthermore, each solvent’s permeance of membrane M3 are higher than M0 due to the transport resistance of solvent molecules in the composite membrane decrease after the doping of MIL-101(Cr). 

**Figure 11:**
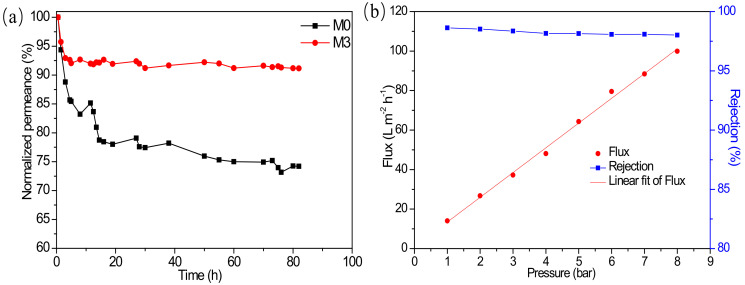
(**a**) Changes to normalized flux over time with the continuous operation; (**b**) OSN performance of the membrane M3 with different operation pressures.

In order to further investigate the operational stability of the membranes, continuous filtration processes were conducted for the membrane M0 and M3 at 4 bar for more than 80 h, in which RB/methanol was employed as the feed solution. As depicted in Figure 11a, the flux of the pristine membranes M0 decreased by 25%, while membrane M3 slightly decreased by 8% at the end of the 80-h test, demonstrating that the doping of MIL-101(Cr) could endow the membrane with superior filtration stability. The reason might be ascribed to the anti-aging effect of MIL-101(Cr) nanoparticles. MIL-101(Cr) can be suddenly wrapped or fixed in the polyamide during the interfacial polymerization between PIP and TMC, and then the MIL-101(Cr) can confine polymer molecular chains inside the internal pores and restrict the migration of polymer chains by the π − π interaction between aromatic rings of MIL-101(Cr) molecules and the polymer matrix [7]. In addition, the flux of the composite membrane M3 maintains an almost linear relationship with the driving pressure (Figure 11b), indicating that the composite membrane did not exhibit membrane compaction under high pressure. In summary, the MIL-101(Cr) modified membrane exhibited improved filtration stability and has satisfied compressive resistance. 

## 4. Conclusions

In summary, we first prepared the PPSU-MoS_2_/PA-MIL-101(Cr) membrane with highly enhanced permeance and stability for organic solvent nanofiltration. By adding a low concentration of MIL-101 (Cr) (0.005 wt% ) to the organic phase for the IP process, the membrane achieved methanol permeance with 12.03 L m^−2^ h^−1^ bar^−1^, more than twice higher than that of the control membrane (5.05 L m^−2^ h^−1^ bar^−1^). Moreover, the optimized composite membrane showed improved operational stability with a very small flux reduction (8%) after 80 h of continuous operation, far exceeding that of pristine membranes. Finally, we are the first to demonstrate that the solvent transmission mechanism of the PA-MIL-101(Cr) composite membrane conforms to the dissolution-diffusion model. 

## Figures and Tables

**Table 1:** The compositions of organic solution for the interfacial polymerization.

Membranes	Organic Solution	
	TMC (w/v%)	MIL-101(Cr) (w/v%)
M0	0.35	0
M1	0.35	0.003
M2	0.35	0.004
M3	0.35	0.005
M4	0.35	0.007
M5	0.35	0.01
M6	0.35	0.02

**Table 2:** The cross-linking degree of membranes M0, M1, M3 and M6.

Membranes	C (%)	N (%)	O (%)	Cr (%)	O/N^a^ (%)	O/N^b^ (%)	Cross-linking Degree ^c^ (%)
M0	67.66	14.55	17.79	-	1.223	1.223	69.91
M1	67.95	14.12	17.90	0.03	1.268	1.255	66.08
M3	67.35	14.05	18.51	0.09	1.317	1.279	63.27
M6	68.73	13.18	18.02	0.07	1.367	1.335	56.92

^a^ The atomic concentration ratio is determined by the XPS. ^b^ After excluding the O atoms in MIL-101 (Cr).^c^ The results were calculated according to the O/N^b^.

**Table 3:** The characteristics of different dyes.

Solute	Molecular-Weight (g mol^−1^)	Chemical Structure	3D Molecuar Structure	Wavelength (nm) MeOH
Rose Bengal (RB)	1017	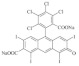	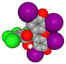	556
Bromothymol blue (BTB)	624	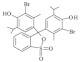	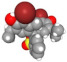	420
Crystal Violet (CV)	408	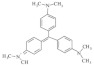	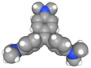	583
Methyl orange (MO)	327	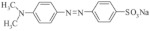	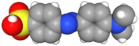	421

**Table 4:** Solvent properties in this experiment.

Solvent	d (nm)	Molar Volume (cm^−3^ mol^−1^)	Viscosity (cP)	Hansen Solubility Parameter (δ, (MPa^1/2^))
n-hexane	86.18	1304	0.307	0
Acetonitrile	-	-	0.302	18
Ethyl acetate	88.1	98	0.44	5.3
MeOH	32	40.7	0.55	12.3
EtOH	46.1	58.5	1.1	8.8
IPA	60.1	76.9	2	6.8

## Data Availability

Not applicable here.
